# The complete mitochondrial genome of the tube-nosed bat *Murina cyclotis* (Chiroptera: Vespertilionidae) in China

**DOI:** 10.1080/23802359.2019.1623125

**Published:** 2019-07-11

**Authors:** Yang Yue, Zhenglanyi Huang, Fang Li, Sanjan Thapa, Yifeng Hu, Yi Wu, Wenhua Yu

**Affiliations:** Key Laboratory of Conservation and Application in Biodiversity of South China, School of Life Sciences, Guangzhou University, Guangzhou, PR China

**Keywords:** Chiroptera, complete mitochondrial genome, Guangxi, *Murina cyclotis*

## Abstract

In this study, the complete mitochondrial genome of a male individual of *Murina cyclotis* from Guangxi province, China, was sequenced and analyzed. The genome is a circular molecule of 16,463 bp length, containing 13 protein-coding genes, 2 ribosomal RNA genes, 22 transfer RNA genes, and a control region. Most of the genes were encoded on the H-strand, except for 8 tRNA and *ND6* genes. Phylogenetic trees of the complete mitochondrial genome were constructed using RAxML. Our result suggests that *M. cyclotis* is closely related to *M. leucogaster* from Korea. The complete mitochondrial genome sequence of *M. cyclotis* will be helpful for future taxonomic and phylogenetic studies on *Murina*.

The species diversity in genus *Murina* is high in Asian region, with 39 recognized species. The current number of species listed is twice the number of species listed until 2005 (Csorba and Bates 2005; Kuo et al. 2006, 2009; Csorba et al. 2007; Kruskop and Eger 2008; Furey et al. 2009; Csorba et al. 2011; Eger and Lim 2011; Francis and Eger 2012; Ruedi et al. 2012; Soisook, Karapan, Satasook, Thong, et al. 2013; Soisook, Karapan, Satasook, Bates 2013; He et al. 2015; Chen et al. 2017). Among these species, *Murina cyclotis* is a medium-sized species. Apparently, this species is similar to *M. huttoni* externally (Francis and Eger 2012; Liu and Wu 2019). However, phylogenetic evidences such as mitochondrial genes (e.g., *COX1*, *Cyt b* and *ND1*) do not support a close relationship between them (Eger and Lim 2011). Surprisingly, the barcoding investigation further indicated occurrences of cryptic species within this taxon (Francis and Eger 2012; Soisook, Karapan, Satasook, Thong, et al. 2013).

In this study, complete mitochondrial genome (MK747248) of a male individual *M. cyclotis* (GZHU 16335) collected from Diding country, Guangxi Province (23°06′51.6″N 105°58′12.6″E), China, was sequenced. Complete genomic DNA was extracted from liver tissue using MiniBEST Universal Genomic DNA Extraction Kit (TAKARA, Dalian) and was sequenced using Illumina Hiseq X.

The total length of the mitochondrial genome is 16,463 bp. It encodes 13 protein-coding genes, two rRNA genes, 22 tRNA genes, and a control region, the same pattern as in the other three *Murina* mitochondrial genomes (Yoon et al. 2013; Yoon and Park 2016; Zhang et al. 2016). All the protein-coding genes (total 11,399 bp) were encoded in the H-strand except for *ND6* in L-strand. Among the 13 protein-coding genes, most common start codon is ATG while the *ND2* and *ND5* start with ATA, *ND3* starts with ATT. Seven protein-coding genes stop with TAA, only *Cyt b* ends with AGA. The rest of five genes stop with an incomplete termination codons TA– (*ND1*, *ND3*) and T–– (*ND2*, *COX3*, *ND4*), which may be modified by the polyadenylation after transcription (Ojala et al. 1981). Total length of the 22 tRNA genes is 1505 bp ranging from 62 bp (tRNA-Ser) to 75 bp (tRNA-Leu) and can be folded into typical cloverleaf secondary structure except for the tRNA-Ser (AGC). The control region with 1018 bp in length is located between the tRNA-Pro and tRNA-Phe genes, and it has high simple repeat sequence GCAATC.

The phylogenetic relationships of *M. cyclotis* were inferred using mitochondrial 13 protein-coding genes of 89 complete mitochondrial genome sequences. PartitionFinder 1.1.1 (Lanfear et al. 2012) was used to select the best partitioning scheme and regional best-fit models of nucleotide evolution. In the phylogenetic tree, all *Murina* species were clustered together, and it revealed closer relationship of *M. cyclotis* with *M. leucogaster* rather than with *M. huttoni*. Besides, *Murina* and *Myotis* clustered more closely together and clearly separated from other genera in Vespertilionidae (Figure 1). Such patterns suggest morphogroups within *Murina* (e.g. ‘*suilla*-group’ and ‘*cyclotis*-group’) do not represent phylogenetic assemblages. Given the fact that a multi-lineages and cryptic species were observed within *M. cyclotis* using DNA barcoding technique (Francis and Eger 2012), future taxonomic and mitochondrial genome sequences throughout its distribution range are desired. Here, the first mitochondrial genome of *M. cyclotis* is provided, and it can benefit future phylogenetic and evolutionary studies on this genus and taxon.

**Figure 1.**
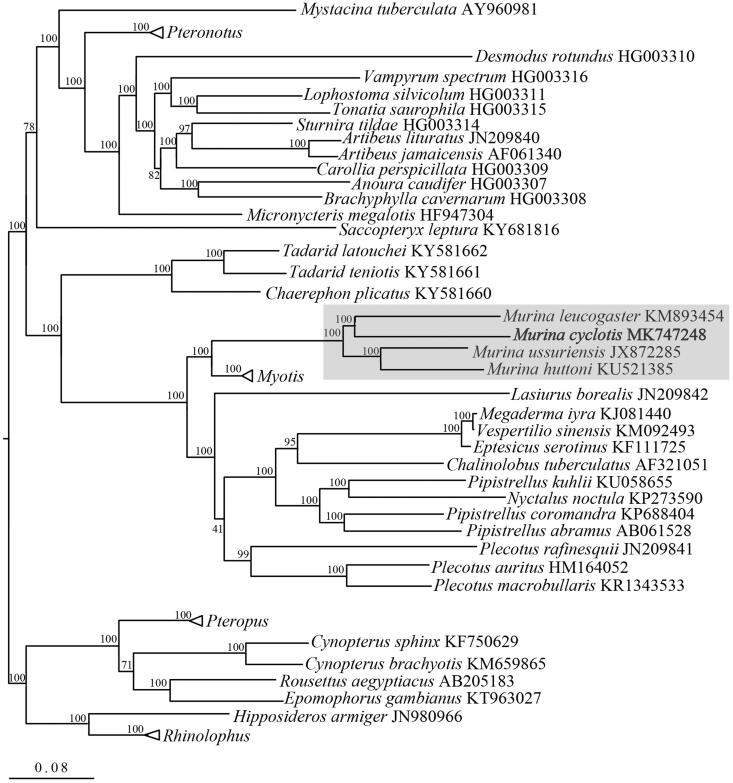
Molecular phylogenetic analysis of 89 species of order Chiroptera (*Pteronotus* contains 4 species, *Myotis* contains 33 species, *Pteropus* contains 4 species, *Rhinolophus* contains 11 species) based on complete mitochondrial genome. The phylogenetic tree was constructed using maximum likelihood method. Percentages of trees where associated taxa were clustered together were shown next to branches, and *Murina* clade highlighted in shade.
